# The Roadmap of Colorectal Cancer Screening

**DOI:** 10.3390/cancers13051101

**Published:** 2021-03-04

**Authors:** Enea Ferlizza, Rossella Solmi, Michela Sgarzi, Luigi Ricciardiello, Mattia Lauriola

**Affiliations:** 1 Department of Experimental, Diagnostic and Specialty Medicine, University of Bologna, 40138 Bologna, Italy; rossella.solmi@unibo.it (R.S.); michela.sgarzi2@unibo.it (M.S); mattia.lauriola2@unibo.it (M.L.); 2 Gastroenterology Unit, Department of Medical and Surgical Sciences, University of Bologna, 40138 Bologna, Italy; luigi.ricciardiello@unibo.it

**Keywords:** fecal immunochemical test (FIT), colonoscopy, flexible sigmoidoscopy, liquid biopsy, mRNA, microRNA, ctDNA, proteins, extracellular vesicles

## Abstract

**Simple Summary:**

Colorectal cancer (CRC) is the third most common form of cancer in terms of incidence and the second in terms of mortality worldwide. CRC develops over several years, thus highlighting the importance of early diagnosis. Fecal occult blood test screening reduces incidence and mortality. However, the participation rate remains low and the tests present a high number of false positive results. This review provides an overview of CRC screening globally and the most recent approaches aimed at improving accuracy and participation in CRC screening, while also considering the need for gender and age differentiation. New fecal tests and markers such as DNA methylation, mutation or integrity, proteins and microRNAs are explored, including recent investigations into fecal microbiota. Liquid biopsy approaches, involving novel markers, such as circulating mRNA, micro-RNA, DNA, proteins and extracellular vesicles are discussed. The approaches reported are based on quantitative PCR methods or arrays and sequencing assays that identify candidate biomarkers in blood samples.

**Abstract:**

Colorectal cancer (CRC) is the third most common form of cancer in terms of incidence and the second in terms of mortality worldwide. CRC develops over several years, thus highlighting the importance of early diagnosis. National screening programs based on fecal occult blood tests and subsequent colonoscopy have reduced the incidence and mortality, however improvements are needed since the participation rate remains low and the tests present a high number of false positive results. This review provides an overview of the CRC screening globally and the state of the art in approaches aimed at improving accuracy and participation in CRC screening, also considering the need for gender and age differentiation. New fecal tests and biomarkers such as DNA methylation, mutation or integrity, proteins and microRNAs are explored, including recent investigations into fecal microbiota. Liquid biopsy approaches, involving novel biomarkers and panels, such as circulating mRNA, micro- and long-non-coding RNA, DNA, proteins and extracellular vesicles are discussed. The approaches reported are based on quantitative PCR methods that could be easily applied to routine screening, or arrays and sequencing assays that should be better exploited to describe and identify candidate biomarkers in blood samples.

## 1. Introduction

Colorectal cancer (CRC) develops over time from modifications of the normal intestinal mucosa to benign precancerous adenomas, carcinoma, and eventually aggressive metastatic cancer [1]. This transition is a complex, multifactorial process that has been characterized over the years. Adenomatous polyposis coli (APC) gene mutations or deletions leading to chromosomal instability represents one of the pathways that drives the development of CRC [2,3,4]. Activating mutations of the KRAS oncogene and inactivating mutations of the TP53 tumor suppressor gene further promote adenoma–carcinoma progression. Microsatellite instability (MSI), aberrant CpG island methylation phenotype (CIMP), chromosomal instability (CIN) and BRAF mutations are also associated with the transition and development of CRC. Colorectal cancer is also linked to different risk factors such as older age, male sex, adverse lifestyle habits (smoking, increased consumption of red meat and alcohol), chronic intestinal diseases, clinical history of polyps, genes, and heredity [5]. 

The slow growth of this cancer makes the identification of precancerous lesions and early detection of cancer fundamental for defeating the disease. Screening is, thus, essential to reduce the incidence and mortality of CRC. In fact, CRC mortality is gradually decreasing in industrialized countries due to the widespread adoption of screening programs [5]. Today, the implementation of screening opportunities is crucial, and the research in this field is prolific globally.

This review is divided in two parts. The first focuses on the CRC status, screening methodologies, national screening programs and their application worldwide. The second part firstly examines the main drawbacks of the fecal occult blood test (FOBT), which is the golden standard screening test worldwide, and then focuses on the strategies aimed at improving CRC screening using liquid biopsy approaches and suitable candidate biomarkers (mRNA, miRNA, ctDNA, microvesicles). 

## 2. Colorectal Cancer Status in Europe and in the World

**Figure 1:**
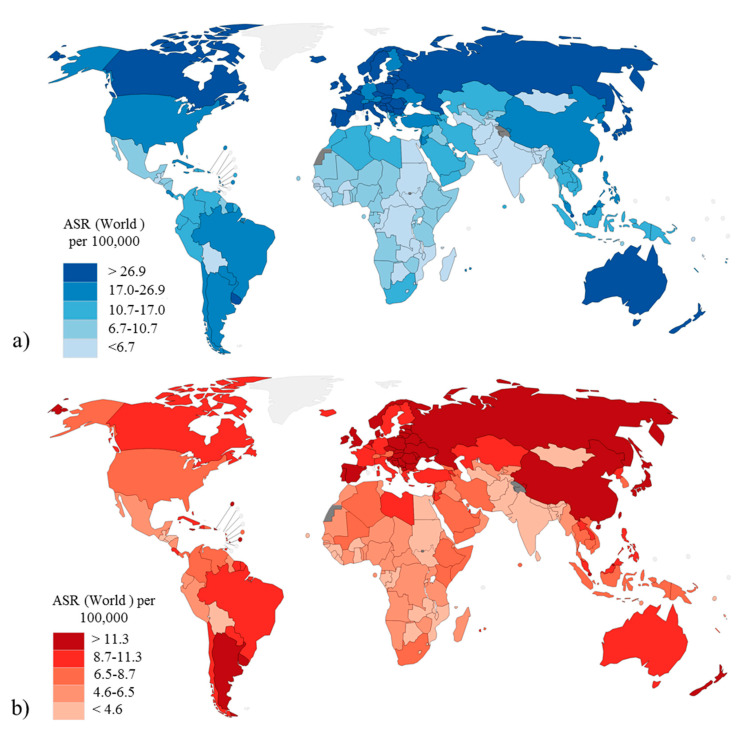
World and colorectal cancer in 2020. (**a**) Estimated age standardized incidence rate (100,000) for world countries; (**b**) Estimated age standardized mortality rate (100,000) for the world countries. Modified from Global Cancer Observatory (GBO) 2020, International Agency for Research on Cancer, World Health Organization [6].

CRC is the third most common form of cancer in terms of incidence and the second in terms of mortality worldwide, with 1.9 million new cases and 930,000 deaths reported in 2020 [6]. There are important geographical discrepancies regarding the incidence and mortality of CRC (Figure 1, Tables S1 and S2). Australia and New Zealand show the highest incidence, followed by Europe and North America [7,8,9,10]. The highest reported mortality rates are in central Eastern Europe. The lowest CRC incidence is registered in South Asia and in Africa, where also the lowest mortality rates are recorded, although in these areas the highest mortality to incidence ratio is recorded.

**Figure 2:**
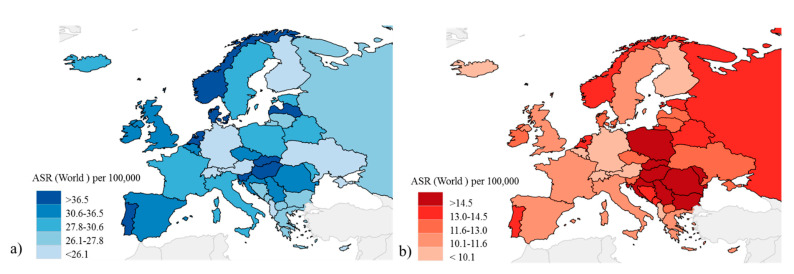
Colorectal cancer in Europe in 2020. (**a**) Estimated age standardized incidence rate (100,000) for European countries; (**b**) Estimated age standardized mortality rate (100,000) for the European countries. Modified from Global Cancer Observatory 2020, International Agency for Research on Cancer, World Health Organization [6].

In Europe, CRC is the second most common oncological disease in terms of incidence and mortality, with 519,820 new cases and 244,824 deaths registered in 2020 [6]. The highest incidence was observed in central Eastern Europe and there are substantial differences between European countries (Table S3; Figure 2), with respect to both risk factors, linked to different lifestyles, and screening policies [5,9,11].

In Italy, CRC is the third most common oncological disease in terms of incidence and the second in terms of mortality, with 49,327 new cases and 19,258 deaths reported in 2018 (Table S4). The 5-year survival is 66%, with no differences between men and women [12]. 

## 3. Colorectal Cancer Screening

### 3.1. Advantages of Screening in Terms of Incidence and Mortality

CRC screening programs have been shown to reduce incidence and mortality [7,13,14,15,16]. Soon after the activation of screening programs, the incidence showed an increase in the short-term, which tended to decrease over the subsequent years [13,17] and the cancers that were detected were more often diagnosed at earlier stages [16]. Notably, populations with active screening programs have shown an impressive reduction in mortality from 22 to 68% [16,17,18,19,20,21].

### 3.2. Screening Tests

#### 3.2.1. Stool-Based Tests

There are currently three types of screening for detecting CRC: stool-based, imaging, and endoscopic tests [5]. Stool-based tests (fecal occult based test, FOBT) shows the presence of hem (gFOBT) or human globin (FIT) of hemoglobin in stool samples. The gFOBT has a long history and consists of a colorimetric assay which uses the guaiac reaction [22]. FIT is an immunochemical test, which exploits a specific antibody. It has replaced gFOBT because it is more sensitive and accurate at detecting CRC (sensitivity 69–95% vs. 25–38%) and does not require dietary restrictions [23,24,25].

Patients with positive FIT tests are referred for a colonoscopy for further investigations. In order to respond to the best cost–benefit strategy, numerous studies have tried to fix the optimal cut-off of FIT. The most commonly used value currently appears to be 100 ng/mL, corresponding to 20 μg of Hb per g of stool [24]. A high variability has been recorded in FIT screening between different centers and kits, with the analytical performance depending on antibody characteristics (mono or polyclonal), buffer volume or composition of collection vials [7,26]. Other drawbacks of FIT are related to the less than optimal level of enrollment in screening programs, the high number of false positive results (15–30%), the poor ability to detect serrated polyps, and the low sensitivity for adenomas [5,7,27].

#### 3.2.2. Imaging Tests

Imaging tests include the double-contrast barium enema (DCBE), computed tomographic colonography (CTC), and colon capsule endoscopy (CCE). Today, novel imaging methods are often used rather than DCBE [5]. CTC was introduced about 20 years ago and provides endoluminal images of the air-distended colon, reconstructed by computed tomography or magnetic resonance [5]. CCE is recognized by the European Society of Gastrointestinal Endoscopy as an acceptable screening method for CRC (with a sensitivity of 84% and specificity of 93%) [28]. However, these methods require intensive bowel preparation and are more expensive than colonoscopy and biopsies cannot be performed [5,28].

#### 3.2.3. Endoscopic Tests

Endoscopic tests consist of flexible sigmoidoscopy (FS) and colonoscopy (CS). FS visualizes only the distal gastrointestinal tract, but does not detect lesions in the proximal colon. The advantages of FS include the fact that no dietary restrictions are required and it involves minimal bowel preparation [5,19,20]. Colonoscopy represents the gold standard for diagnosis, with a high sensitivity and specificity for detecting cancerous and precancerous lesions (97–98%) in the entire large bowel and the distal part of the small bowel [18]. During the procedure, it is also possible to perform biopsies for histological evaluation. However, colonoscopy is an expensive and risky method, since complications such as bleeding or bowel perforation occur in approximately 0.1–0.2% of patients [5,7,19].

### 3.3. Screening Status in Europe and the World

In 2012, the European Union drew up guidelines on CRC screening and diagnosis, recommending the use of national screening programs based on FIT, FS or CS [29].

In Italy, the national screening program is FIT-based, which is recommended every two years and carried out for the population deemed at risk (50–69 years) [7,12,30,31,32]. According to the most recent data, screening coverage was between 90 and 96%, depending on the geographic area; however, the overall participation was still low, ranging from 60% in the north to 23% in the south (Tables S5 and S6). 

Several European countries, including the extra-EU, have developed an ongoing or planned national or regional screening program, with an invitation system for the population considered at risk (Table 1). The majority use the fecal occult blood test (gFOBT or FIT), with wide differences in participation rates. 

National and regional organized screening programs using the fecal test (gFOBT or FIT) have also been reported for Canada, Brazil, Argentina, Chile and Uruguay, which obtained high participation rates in the pilot studies (90.1–79.7%) [10,36].

On the other hand, in the USA, the U.S. Preventive Service Task Force recommends that asymptomatic adults aged from 50–75 have a screening test on a voluntary basis, and a national screening program is still not available. The choice for the average risk population in the USA is between stool-based tests (gFOBT, FIT, FIT-DNA), direct visualization tests (FS, CS, CTC) or the serological DNA test (SEPT9). As of 2018, 68.8% of people aged 50–75 with health insurance are reported to be up-to-date with colorectal cancer screening [40]. 

In 2015, recommendations for CRC screening in the Asia Pacific region were updated [41] and the Asia Cohort Consortium focused also on health outcomes in Asian populations [42]. Few countries (Australia, China, Japan, New Zealand, South Korea, Thailand and Taiwan) have national or regional screening programs and these are mainly based on the fecal test (FIT or gFOBT). Additionally, in these regions the national participation rates were rather low (13–41.3%) [10,36].

Among the countries in the eastern Mediterranean, only Israel reported an organized screening program based on FIT designed for people aged 50–74 years, while in other countries, only opportunistic screening has been adopted (Jordan, Qatar and the United Arabic Emirates). Finally, regarding Africa, the adoption of organized screening may have a limited impact due to the relatively low incidence of CRC and the limited economic resources [10,36].

## 4. Disadvantages of Fecal Tests (FIT/gFOBT), Room for Improvement

In order to fully benefit from screening programs, the participation rate should be higher than 80%, thus, low take-up is one of the main drawbacks of all the screening programs, with large differences among and within countries [10,16,17,19,31,36,43,44]. Numerous studies have addressed the pitfalls of FIT, including the high rate of false positives and negatives. A false positive FIT can create unnecessary psychological distress and superfluous requests for colonoscopies, with associated healthcare costs. Between 8 and 32% of FIT positive participants do not have significant lesions [19,45,46]. On the other hand, false-negative FIT results can delay CRC diagnosis and dissuade participants from subsequent evaluations [47,48,49]. 

The FIT sensitivity for CRC ranges from 91 to 71%, according to the Hb cut-off, with specificity ranging from 90 to 95%. For advanced adenoma (AA), the sensitivity falls from 40 to 25%, with specificity ranging from 90 to 95% [50,51]. There are several risk factors for CRC: gender, age, obesity, alcohol consumption, current or former smoking and the use of drugs, such as non-steroidal anti-inflammatory drugs (NSAIDs) or anticoagulants [24,27,47]. Differences in FIT performance by sex and age have been described. The pooled sensitivity of FIT for advanced neoplasia (AN) was higher in women than in men, with pooled specificities of 92 and 94%, respectively. Accordingly, De Klerk et al., found that the highest risk of a false positive was found for females and the use of NSAIDs [47]. Interestingly, low-dose aspirin was associated with a higher risk of false positives (FPs), suggesting a possible effect on bleeding of early lesions. Several factors appear to be associated with an increased risk of false-positive FIT: male sex, older age (>65), obesity, and current smoking [27,52].

The importance of improving FIT screening has led to various strategies aimed at finding a balance between resources, participation rates and large populations. Some countries have decided to increase the FIT positivity threshold, with the hope of reducing the false positive results and optimizing colonoscopy performance. However, a high threshold leads to a decreased sensitivity and an increased specificity only for advanced neoplasia [53]. This solution reduces the number of false positives, but also increases the false negatives. The decision to increase the FIT cut-off value “simply” to reduce the number of colonoscopies seems more “cost-effective” than “patient-effective”, and the adjustment of the FIT cut-off value cannot be the only viable solution. 

A novel approach to FIT screening was developed by Senore et al., who evaluated the sum of quantitative FIT results during consecutive negative screening rounds [54]. Subjects with a cumulative fecal Hb level ≥20 µg/g showed an 18-fold increase in their cumulative AN (CRC and AA) risk over the subsequent two rounds. This is an interesting approach; however, the number of false positive FITs would still be very high. Another option is to select gender-specific or age-specific cut-offs in FIT screening [53,55,56]. In a stratification model, patients could be assigned to different risk levels of finding AN, by combining different risk factors (such as sex, age or Hb value), or considering FIT separately from the prediction model. The risk-stratification based on prediction models might be better at predicting neoplastic outcomes, including all FIT results, and might enlarge the eligible population including younger subjects (<50 years) and/or people with a history of familiarity for CRC. 

## 5. New Tests

### 5.1. Fecal Tests

In recent years, new tests have been developed to optimize CRC screening and diagnosis. Since the first studies on *RAS* oncogene mutations [57], DNA alterations in stools have been investigated, as well as proteins and microRNAs.

A recent systematic review evaluated the performance of FIT combined with other stool markers, including DNA methylation, mutation or integrity markers (PHACTR3, APC, p53, KRAS and BRAF), proteins (transferrin, calprotectin and calgranulin) and microRNA (miR-106a) [58]. Notably, the largest increase in sensitivity for CRC was found with long DNA as a measure of DNA integrity in the APC gene and p53, with a specificity of 98%. Among the protein markers, combining transferrin or calgranulin C tests to FIT yielded a slight increase in sensitivity [58,59]. However, calprotectin led to a significant increase in sensitivity for all adenomas, from 53 to 86%, but the specificity decreased from 68 to 26% [58,60].

Following the first studies on DNA and the feasibility of the prototype of a multitarget panel assay in stools [61], Imperiale et al. further developed and tested the multitarget stool DNA ColoGuard (MT-sDNA; Exact Science) in a large screening setting. This test is currently an alternative stool test, which was approved by the Food and Drug Administration (FDA) and is currently employed in the USA [62]. The MT-sDNA test, ColoGuard, combining stool DNA markers (methylated BMP3 and NDRG4 promoter regions, mutant KRAS) with the results of FIT, undoubtedly represented a milestone of stool-based molecular testing in CRC screening. It was tested in nearly 10,000 people, showing a significantly better sensitivity than FIT in predicting any stage of CRC (92.3 vs. 73.8%, respectively) and AA (42.2 vs. 23.8%), and retaining a high specificity (89.9 vs. 96.4%). Similar results were obtained by Bosch et al., in a cohort of 1014 people [63]. By comparing the single-application performance of the MT-sDNA test with FIT, MT-sDNA showed a greater sensitivity for AA than FIT at the lowest cut-off tested (10 µg Hb/g of feces), with a slight decrease in specificity (94 vs. 98%). No significant difference was highlighted to distinguish between proximal and distal AA.

A similar FIT-DNA test kit, ColoClear, manufactured by New Horizon Health Technology Corporation Limited in Hangzhou, China, calculates a risk prediction by combining the FIT test with the detection of the KRAS gene mutation, NDRG4 and BMP3 methylation. ColoClear was tested in 839 subjects, obtaining a sensitivity for CRC and AA of 97.5 and 53.1%, respectively, with a combined sensitivity for predicting AN (CRC and AA) of 88.9% and a specificity of 89.1% [64]. Moreover, no significant difference was highlighted for proximal or distal colon CRC, while sensitivity for distal AA was higher than for proximal AA (61 vs. 30%).

Another stool test, developed in Italy, with the collaboration of Diatech Pharmacogenetics, is based on the evaluation of stool DNA integrity [65,66]. The authors carried out a quantitative evaluation based upon fluorescence amplification of different genomic DNA targets called fluorescence long DNA (FL-DNA). FL-DNA showed a 70% sensitivity and 87% specificity in detecting CRC in stool samples from subjects recruited by a regional screening program based on FIT positivity. 

Microbiome-based tests could represent a new frontier in the CRC detection. Grobbee et al. measured fecal microbiota in FIT positive subjects. An overall increase in total bacterial content (16S) was associated with patients affected by high grade dysplasia and CRC [67]. Similarly, a new non-invasive CRC screening test based on microbiome data was employed to reduce the false positive rate of FIT [68]. The authors targeted specific genomic DNA bacterial sequences: Eubacteria (EUB) as the total bacterial load, *Faecalibacterium prausnitzii* (B10), *Subdoligranulum variabile* (B46), *Ruminococcus*, *Roseburia* and *Coprococcus* (B48), *Roseburia intestinalis* (RSBI), *Gemella morbillorum* (GMLL), *Peptostreptococcus stomatis* (PTST), *Bacteroides fragilis* (BCTF), *Collinsella intestinalis* (CINT), and *Bacteroides thetaiotaomicron* (BCTT). GMLL, PTST and BCTF correlated significantly with AN. Although the sensitivity values for bacterial markers alone were much lower than FIT performance, a final algorithm consisting of the combination of FIT with three ratios between bacterial markers (PTST/EUB, BCTF/EUB, BCTT/ EUB) decreased the number of false positive results by 50%, obtaining a sensitivity of 80% and a specificity of 90%. Finally, panels of proteins were tested in stool samples to identify AA or AN subjects, however, the levels of sensitivity and specificity were quite low (54 vs. 13%) (Hp, LRG1, RBP4, and FN1; 62 vs. 40%) [69].

Nevertheless, despite these efforts to improve fecal-based screening, the main drawbacks remain the low participation rate and the costs [70,71]. 

### 5.2. A New Alternative: Liquid Biopsy

The analysis of tumor-derived biomarkers in biological fluids has the potential to increase the participation rate [72]. Peripheral blood is one of the most studied biological fluids, and an accurate blood test could be an attractive alternative for asymptomatic, average-risk individuals who are reluctant to undergo screening by a stool test or endoscopy. Two independent surveys [73,74] showed that a blood sample would be preferred to a stool sample in a screening setting. In a clinical trial, 12% of people who refused to enroll in a stool-based screening, agreed to perform the blood-based test [71]. Thus, blood CRC biomarkers remain very attractive and are under investigation, including several molecules from nucleic acids such as DNA and various types of RNA (messenger, mRNA; micro, miRNA; long non-coding, lncRNA) to proteins, from circulating tumor cells to microvesicles. There is also growing interest in biomarker combination, which could obtain a higher sensitivity than single biomarker-based tests [72,75,76].

**Figure 3:**
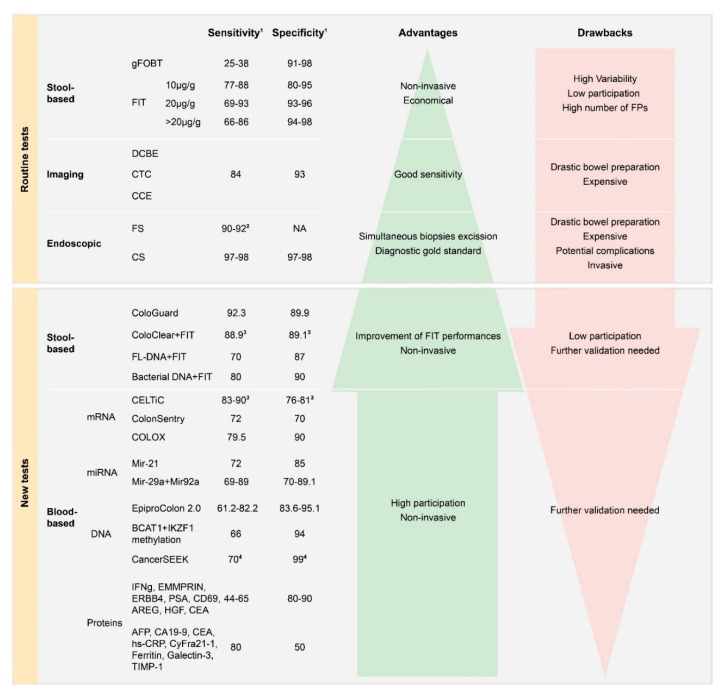
Routine and new tests used or proposed for colorectal cancer screening. Guaiac fecal occult blood test (gFOBT), fecal immunochemical test (FIT), double-contrast barium enema (DCBE), computed tomographic colonography (CTC), colon capsule endoscopy (CCE), colonoscopy (CS), flexible sigmoidoscopy (FS), fluorescent long DNA (FL-DNA), not available (NA), false positive (FP). ^1^ The reported percentages of sensitivity and specificity refer to colorectal cancer. ^2^ Refers only to distal colorectal cancer. ^3^ Refers to advanced neoplasia. ^4^ Refers to the average value among 8 cancer types.

Figure 3 summarizes the blood-based tests discussed in this review. The next part of this review focuses, above all, on mRNA, including microRNAs and lncRNA. DNA, proteins and microvesicles are also briefly discussed.

#### 5.2.1. mRNA

The emergence of RNA sequencing (RNA-seq) technologies with the evolution of next generation sequencing (NGS) is promising for the diagnosis, prognosis and therapy of cancers including CRC. However, this method is very expensive and, paradoxically, provides too much information that is not yet fully exploitable for the purpose of early diagnosis [77].

In 2016, Rodia et al. [78] used a novel bioinformatic approach to search for specific RNAs with high differential gene expression between CRC and normal blood. The genes showing the highest significant difference were analyzed by qRT-PCR in blood samples of healthy and CRC patients. The authors reported that *CEACAM6*, *LGALS4*, *TSPAN8* and *COL1A2* (known as CELTiC) discriminated between the two groups with a sensitivity and specificity of 92 and 67%, respectively. The CELTiC panel was subsequently analyzed in a population of FIT positive subjects, confirming its ability to identify patients with high-risk lesions (CRC and AA), and appeared able to discriminate false positive FIT and low risk patients (non-advanced adenoma and polyps) [79]. In 2020, the CELTiC panel was measured in blood samples from healthy FIT negative subjects, reporting significant gender differences for *CEACAM6* and *COL1A2*, thus highlighting the importance of gender as a potential factor in the comparison between healthy and FIT false positive subjects. The CELTiC panel obtained high AUCs when comparing healthy to AN, low risk, FIT false positive subjects or a combination of these groups, with good sensitivities and specificities ranging from 83 to 90% and from 76 to 81%, respectively. These results confirmed the need for additional studies to better define gender- and age-specific reference intervals for the early diagnosis of CRC [80]. 

A similar approach was applied using ColonSentry, a panel of seven mRNAs [81,82]. Six out of seven genes (*ANXA3*, *CLEC4D*, *LMNB1*, *PRRG4*, *TNFAIP6* and *VNN1*) were overexpressed in the blood of CRC patients, and one (*IL2RB*) was under expressed with a blinded validation test set resulting in 72% sensitivity and 70% specificity, with similar predictive values for left- and right-sided CRC.

A similar test is COLOX [83,84], a panel of 29 mRNAs (BCL3, IL1B, PTGS2, MAP2K3, PTGES, PPARG, MMP11, CCR1, EGR1, CACNB4, CES1, IL8, S100A8, CXCL11, ITGA2, NME1, JUN, TNFSF13B, CXCR3, MAPK6, CD63, ITGB5, GATA2, LTF, MMP9, CXCL10, MSL1, RHOC, FXYD5) measured in the peripheral blood mononuclear cells. Few individual genes showed significant differences among age classes, but the whole panel was not affected by the age of the patient. Twelve mRNAs (BCL3, IL1B, PTGS2, PTGES, PPARG, MMP11, CCR1, EGR1, CACNB4, CES1, IL8, S100A8) were able to differentiate between the control group and CRC, and five mRNAs (CES1, CXCL11, IL1B, ITGA2, NME1) identified large adenomas. The authors also found seven markers specifically able to differentiate between large adenomas and CRC (BCL3, PTGES, PPARG, MMP11, IL8, TNFSF13B, CXCR3). 

Other mRNAs have also been investigated as blood markers of CRC [85,86,87]. In 2018, Alamro et al. reported significantly higher mRNA expression of inflammatory genes (COX-2, TNF-α, NF-κB, IL-6) in blood samples of 20 CRC compared to 15 healthy controls without significant association with gender, age or tumor localization [85]. A case–control study performed on 83 CRC patients and 11 healthy donors resulted in significantly higher levels of circulating HMGA2 mRNA in CRC patients with an AUC of 0.932 and a sensitivity of 86.8%. The authors highlighted also a significant association with tumor localization, reporting a greater expression in patients with colon cancer and right-sided CRC, but not with age or gender of the patients [86]. Hamm et al. performed a genome-wide expression analysis on RNA obtained from peripheral blood monocytes collected from 329 subjects (128 healthy, 160 CRC, 41 other gastric diseases) divided in different cohorts [87]. Twenty-three genes showed differential expression between healthy and CRC. By testing different statistical models, the authors reported sensitivity values from 80 to 100%, specificity from 92.3 to 93.3%, and AUCs from 0.86 to 0.99. However, the panel was not tested for the evaluation of preneoplastic lesions (i.e., polyps) or tumor localization [87].

#### 5.2.2. miRNA

MicroRNAs (miRNAs) are small non-coding RNAs (~20–22 nucleotides) that regulate gene expression through repression or degradation of mRNAs. miRNAs seem to be promising plasma biomarkers associated with the onset of CRC, and several studies have searched for specific panels of miRNA capable of increasing both the sensitivity and specificity of screening [4,7,88,89,90,91]. miR-7, miR-17-3p, miR-18, miR-21, miR-29a, miR-31, miR-92a, miR-93, miR-155, miR-181b, miR-200c, miR-221, miR-409-3p, let-7g are some of the miRNAs tested, either individually or as a panel in plasma or serum of patients affected by CRC. However, only a few have been confirmed as diagnostic CRC biomarkers by more than one study [89,90,91,92,93]. Among the most studied, mir-21 and mir-29 family (mir-29a, mir-29b and mir-29c) are overexpressed in CRC and associated with CRC progression and metastasis [7,91]. miR-21 is one of the most investigated diagnostic markers of CRC, identified in several biological fluids (plasma, serum, whole blood) [72,94]. 

Clusters of miRNA have been taken into consideration and the mir-17–92 cluster, also called oncomiR-1, is one of the most studied clusters in relation to CRC. This cluster contains different members such as miR-17, miR-18a, miR19a, miR-19b, miR-20a and miR-92a, and evidence suggests that miR-29a and miR-92a may have a good sensitivity (69 to 89%) and specificity (70 to 89.1%) in CRC detection. In addition, the combination of miR-92a and miR-29a appears to increase the performance of single miRNAs for detecting AN [75,91,95]. Other members, such as miR-19a and miR-19b, were upregulated in plasma from CRC patients compared to healthy individuals, and their combination obtained an AUC of 0.82. The combination with another four plasma miRNAs (miR-18a, miR-29a, miR-15b and miR-335) showed promising results in differentiating between controls and CRC (AUC 0.95) or AA (AUC 0.91) and with similar performances for proximal (AUC 0.97) and distal (AUC 0.95) CRC [75,96]. These results highlight the importance of combinatorial approaches involving specific panels of miRNAs. Other panels of miRNA have recently been reported [97,98,99]. A panel of seven miRNAs (miR-103a-3p, miR-127-3p, miR-151a-5p, miR-17-5p, miR-181a-5p, miR-18a-5p and miR-18b-5p) was identified and evaluated in a four-stage experiment (screening, training, testing and external validation) involving a total of 139 CRC patients and 132 controls. The performances of the panel obtained an AUC of 0.895 with a sensitivity and specificity of 76.9 and 86.7%, respectively, without significant associations between serum levels of the analyzed miRNAs and age, gender or location [99].

However, the use of mRNA and miRNA is still limited due to the lack of extensive clinical validations.

#### 5.2.3. DNA

In addition to RNA, DNA has also been widely studied in liquid biopsies searching for CRC biomarkers. Cell-free DNA (cfDNA) and the tumor-derived fraction termed as circulating tumor DNA (ctDNA) are of great interest. cfDNA mutations in genes frequently associated with tumorigenesis have been assessed for the early detection of the most common tumor types, including CRC [72,100,101]. KRAS mutations were detected in plasma from CRC patients, however it was also reported that 0.45–20% of healthy individuals may carry genomic alterations in cfDNA, with particular regard to TP53 and KRAS variants [72]. The low abundance of tumor-derived DNA is one of the main challenges for early detection, as well as cancer-associated mutations accumulated with age. Aberrant DNA methylation is a feature of most solid cancers and is a promising biomarker for early diagnosis [75,100,102]. Septin 9 (SEPT9), a GTP-binding protein belonging to the Septin family, is one of the most widely studied DNA markers in blood in relation to CRC. In fact, CRCs show an atypical methylation status of SEPT9 gene. The EpiProcolon assay, which detects circulating methylated SEPT9 (mSEPT9), was recently approved by the FDA [102,103]. The values of sensitivity obtained from independent studies for mSEPT9 ranged from 48.2 to 95.6%, with specificity ranging from 79.1 to 99.1%. In the most recent studies, the sensitivity of the EpiproColon test 2.0 ranged from 61.2 to 82.2% and the specificity from 83.6 to 95.1%, showing a better performance than carcinoembryonic antigen (CEA) and/or FIT tests in the screening of asymptomatic populations [75,102,103,104]. However, mSEPT9 is not able to distinguish between CRC and polyps or adenomas and seems not affected by tumor localization, but may be affected by age or sex, suggesting that age- and sex-specific cut-offs are required to better optimize the screening and diagnostic procedures [75,102,104]. The combination of mSEPT9 with the FIT test seems to improve the sensitivity for CRC and AA detection obtaining 94 and 43%, respectively, but at the cost of losing the specificity [76].

Other ctDNA markers, such as BCAT1 and IKZF1, have been studied. BCAT1 and IKZF1 methylation obtained a sensitivity of 66% and a specificity of 94% for CRC detection in a prospective study analyzing more than 2000 individuals, including 129 people with CRC [75,105]. A different approach was recently applied by Cohen et al. [106] who described a multi-analyte test (CancerSEEK) to identify eight common cancers, including CRC, by determining the levels of circulating proteins and mutations in ctDNA. The median sensitivity of CancerSEEK was 73 and 78% for stage II and III cancers, respectively, and 43% for stage I cancers. In particular, 14 out of the 16 genes tested (AKT1, APC, BRAF, CDKN2A, CTNNB1, FBXW7, FGFR2, GNAS, KRAS, NRAS, PIK3CA, PPP2R1A, PTEN, TP53) were detected in plasma samples of CRC patients. CRC was also the type of cancer detected with the highest prediction accuracy. 

Another approach [107] analyzed cfDNA and ctDNA by applying targeted error correction sequencing (TEC-Seq) for the sensitive and specific detection of low-abundance sequence alterations using NGS in commonly altered cancer genes. In plasma samples from 44 healthy individuals and 194 patients affected by CRC (n = 42), lung (n = 65), ovarian (n = 42) or breast (n = 45) cancers, the authors analyzed a panel of 55 cancer driver genes. cfDNA was significantly higher in cancer patients than in healthy individuals and, within CRC patients, stage IV showed significantly higher cfDNA than stages I to III. In addition, 83% of CRC patients had detectable alterations in driver genes (ctDNA). These detection rates were higher in patients with stages II, III and IV, (89, 90 and 94%, respectively) and were also detected in half of the patients with stage I cancer, suggesting that larger panels of ctDNA may improve the ability to detect small tumors and pre-neoplastic lesions. 

cfDNA in the blood samples of CRC patients has also been studied using NGS, machine-learning approaches, genome sequencing and digital sequencing technologies [108,109,110,111]. The various models applied by Wan et al. [108] obtained a variable sensitivity (71–85%) with 85% specificity, showing promising preliminary results.

#### 5.2.4. Proteins

Carcinoembryonic antigen (CEA) and carbohydrate antigen (CA19-9) are two of the most studied gastrointestinal tumor-associated proteins in blood (or plasma/serum) [90,112,113,114]. Serum CEA and/or CA19-9 levels are significantly higher in CRC patients compared to healthy subjects and are well-known cancer markers. However, CEA and CA19-9 concentrations may also be high in other conditions or tumors and their usefulness as CRC screening biomarkers is still an open issue. However, today CEA and CA19-9 are used and approved in clinical practice to detect metastatic disease, recurrence, or to monitor response to treatments [114,115,116,117,118,119,120].

Proteomic approaches have recently been applied to blood samples (or plasma/serum) of CRC patients to search for new biomarkers in screening or diagnosis [121,122,123,124]. Chen and colleagues performed protein profiling quantifying tumor-associated protein biomarkers in CRC and healthy control plasma samples. Seventeen proteins showed significantly different concentrations between CRC and controls, nine were overexpressed (CEA, GDF-15, AREG, IL-6, CXCL10, CXCL9, PSA, TNFα, cathepsin-D), and eight were downregulated (HGF receptor, CXCL5, ERBB4, FLT3L, CD69, EMMPRIN, VEGFR-2, Caspase-3). Carcinoembryonic antigen (CEA), growth differentiation factor 15 (GDF-15), and amphiregulin (AREG) were the most significant. In addition, applying a logistic regression model, the authors constructed a multi-marker prediction algorithm including eight markers (IFNg, EMMPRIN, ERBB4, PSA, CD69, AREG, HGF receptor and CEA) reporting moderate sensitivities (44–65%) at high specificities (80–90%) [117].

Finally, an additional panel of eight plasma proteins including AFP, CA19-9, CEA, hs-CRP, CyFra21-1, Ferritin, Galectin-3 and TIMP-1 was tested in 4698 subjects including CRC, AA, non-advanced adenomas and extracolonic cancers [115,125]. All the individual biomarkers significantly identified AN (CRC + AA) and the multivariable model including all the biomarkers and age and gender obtained an AUC of 0.76, with 80% sensitivity and 50% specificity. However, the various models tested, including a combination of the eight proteins, showed moderate performances (90% specificity, 19% sensitivity) in discriminating AA from other conditions (non-advanced adenomas, non-colonic tumors, healthy).

#### 5.2.5. Extracellular Vesicles

Extracellular vesicles (EVs), such as exosomes (EXOs), microvesicles (MVs) and large oncosomes, may contain promising biomarkers. Three main categories divide EVs on the basis of biogenesis and approximate size: EXOs (~40–100 nm) derive from multivesicular bodies within the cells; MVs (~100 nm–1 µm) are formed from the outward budding of the plasma membrane; apoptotic bodies (APs) (~1–5 µm) arise from dying cells undergoing apoptosis [72]. In addition to these classes, some cancer-specific subtypes of EVs have been identified: oncosomes (~100–400 nm) produced by non-transformed cells, whose contents can determine oncogenic effects, and large oncosomes (~1–10 µm) derived from malignant cells [126]. EVs contain proteins, RNA, DNA and lipids, which reflect in part the composition of the cell of origin. By protecting nucleic acids from degradation, EVs could also be considered a better source for tumor molecular profiling compared with cell-free nucleic acids [126,127]. EVs and EXOs are also secreted by cancer cells and in a greater amount than normal cells, therefore, increasing the transfer of RNAs, growth factors and chemokines participating in cancer progression [128,129,130]. Examples of molecules identified in EVs at increased levels include surface proteins detected by flow cytometry, such as the epithelial cell adhesion molecule (EpCAM), CD9, CD81, CD63 and CD147 in the bloodstream of CRC patients [126,131,132,133]. 

One of the drawbacks of studying EVs and exosomes is the lack of a standardized protocol to isolate them from blood and to extract their content or surface material [133,134,135,136,137]. Despite the differences in EV isolation and although most of the studies are case–controls, miRNA is one of the classes most studied as a biomarker in EVs. 

Ogata-Kawata et al. [138] evaluated 88 CRC patients and 11 controls to assess the ability of serum EV-miRNAs. In particular, miR-21, miR-23a, and miR-1246 differentiated CRC patients (all stages) from controls [138]. By comparing serum EV-miRNA from CRC patients to healthy controls, Yan et al., found that miR-486 was significantly upregulated, while miR-548c was significantly downregulated [139]. Liu et al. also reported an increase in miR-486 levels in the serum EVs of CRC patients compared to healthy subjects [140]. Peng et al. further confirmed the downregulation of serum EV miR-548c in CRC patients, also finding an association with shorter survival and liver metastases [141]. 

Other authors have evaluated panels of EV-miRNA. Min et al. [142] analyzed EV-miRNA from blood samples of early-stage CRC patients and non-cancerous controls. The authors found 38 miRNAs upregulated and 57 downregulated in CRC patients compared to healthy controls, some of which, such as Let-7b-3p, miR-150-3p, miR-145-3p, miR-139-3p, had already been reported in the plasma of CRC patients. ROC curve analysis of the single miRNAs reached AUCs of 0.792, 0.686, 0.692, and 0.679, respectively. On the other hand, a logistic model including let-7b-3p, miR-139-3p, and miR-145-3p, confirmed the increased potential of panels of EV-miRNAs compared to individuals ones with an AUC of 0.927 [142]. 

Cha et al. [143] evaluated eight mRNA markers (MYC, VEGF, CDX2, CD133, CEA, CK19, EpCAM, and CD24) extracted from plasma EVs. Of the eight mRNAs, the combination of VEGF and CD133 showed statistically significant differences between healthy and CRC, and obtained an AUC of 0.96 with 100% sensitivity and 80% specificity in discriminating between the two groups. 

EVs also contain other types of RNA, such as long non-coding RNA (lncRNA) or mRNA which are dysregulated in CRC. The presence and performance of lnc-RNA and mRNA has been assessed in APs, MVs and EXOs [144]. In a first screening, in sera of CRC patients and healthy subjects, 21 lncRNAs and 16 mRNAs showed significant differences between EXOs of healthy and CRC samples. In the subsequent validation phase, tested in 30 CRC, 20 adenoma and 30 healthy subjects, the combination of lncRNA breast cancer anti-estrogen resistance 4 (BCAR4) with two mRNAs (keratin-associated protein 5–4, KRTAP5-4, and melanoma antigen family A3, MAGEA3) provided the greatest predictive ability, with an AUC of 0.877. 

## 6. Conclusions

There is a long history of CRC screening tests and several studies have attempted to discover cancer biomarkers in stool or blood samples. However, most of the identified biomarkers, (mRNAs, miRNAs, ctDNA, EVs) have only been evaluated in preliminary case–control studies. 

In order to improve the screening and the diagnosis of CRC, large-scale randomized studies are needed to confirm the clinical benefits and the usefulness of these tests. In particular, RNA-seq and NGS, could be used to describe and characterize the evolution and development of CRC, in order to discover new and earlier biomarkers, thus improving outcomes. On the other hand, qRT-PCR may be simpler and cheaper when applied to panels of biomarkers aimed at higher levels of performance in terms of sensitivity, specificity, accuracy and speed of execution. 

In addition to the differences in sensitivity and specificity between tests, and sometimes the lack of extensive investigating trials, the main drawback remains the low participation rate. However, the use of blood samples may change this trend. Liquid biopsy could be also used to assess the prognosis, response to therapies and during follow-up.

Finally, the development of algorithms, including those derived with artificial intelligence, which associate outcome-influencing parameters such as gender and age with candidate markers, will be a further tool to improve the current efficacy of CRC screening.

## Figures and Tables

**Table 1:** Colorectal cancer (CRC) screening in Europe [15,33,34,35,36,37,38,39].

Country	Program	Test ^1^	Cut-Off ^2^	Target Age ^3^	Interval ^3^ (years)	Invited ^4^ (%)	Participation ^5^ (%)
Albania	NA	NA	NA	NA	NA	NA	NA
Austria	Regional/Opportunistic	FIT/gFOBT/CS	NA	40–80	1	NA	NA
Belarus	NA	NA	NA	NA	NA	NA	NA
Belgium	Regional	FIT/CS	15	50–74	2	99.2	27.7
Bosnia and Herzegovina	Regional/Opportunistic	gFOBT	NA	>50	NA	NA	NA
Bulgaria	No/Opportunistic	gFOBT	NA	NA	NA	NA	NA
Croatia	National	gFOBT	NA	50–74	2	100	15.3
Cyprus	Pilot/Planned	FIT	NA	50–69	2	NA	NA
Czech Republic	Regional/Opportunistic	FIT/gFOBT/CS	15	50–79	2	NA	22.7
Denmark	National	FIT	20	50–74	2	25	64
Estonia	Pilot/Planned	FIT	NA	60–69	2		
Finland	Pilot/Planned	gFOBT	NA	60–69	2	23.9	66.4
France	Regional	FIT/gFOBT	30	50–74	2	99.1	26.5
Germany	Opportunistic/Pilot/planned	FIT/gFOBT/CS	NA	50–74	2–10	NA	NA
Greece	No/Opportunistic	gFOBT/CS	NA	50–74	NA	NA	NA
Hungary	Pilot/Planned	FIT	20	50–70	2	21.1	36.7
Iceland	Opportunistic/Planned	gFOBT/CS	NA	55–75	2–10	NA	30
Ireland	National	FIT	20	60–69	2	10.9	39.6
Italy	National	FIT/FS ^6^	20	50–74 ^6^	2	75	42
Latvia	No/Opportunistic	gFOBT	NA	50–74	NA	NA	11.1
Lithuania	Opportunistic/Pilot/Planned	FIT	NA	50–74	2	NA	53.1
Luxembourg	Opportunistic/Planned	FIT/gFOBT/CS	NA	55–74	2	NA	NA
Macedonia	No/Opportunistic	FIT	NA	NA	NA	NA	NA
Malta	National	FIT	16–20	55–66	2	100	45.4
Montenegro	Regional	FIT	NA	50–74	2	NA	33.3
The Netherlands	National	FIT	47	55–75	2	38.5	71.2
Norway	Regional/Pilot	FIT	NA	55–64	2	NA	64.8
Poland	National	CS	NA	55–64	10	12.5	16.7
Portugal	Regional	FIT/gFOBT	20	50–70	2	1.6	62
Romania	No/Opportunistic	NA	NA	NA	NA	NA	NA
Russian Federation	Opportunistic/ Pilot	FIT/CS	NA	48–75	NA	NA	NA
Serbia	National	FIT	NA	50–74	2	NA	58.4
Slovakian Republic	No/Opportunistic	FIT/gFOBT/CS	NA	NA	NA	NA	NA
Slovenia	National	FIT	20	50–69	2	93	47.1
Spain	National	FIT/gFOBT	20	50–69	2	14.2	50.2
Sweden	Regional	gFOBT	NA	60–69	2	100	62.7
Switzerland	No/Opportunistic	FIT/CS	NA	50–69	2–10	NA	22
Ukraine	NA	NA	NA	NA	NA	NA	NA
United Kingdom	National	FIT/gFOBT/FS	NA	50–74 ^6^	2	100	56.1

^1^ Guaiac fecal occult blood test (gFOBT), fecal immunochemical test (FIT), colonoscopy (CS), flexible sigmoidoscopy (FS), not available (NA). ^2^ Cut-off for FIT in µg Hb/g feces ^3^. Target age and interval screening according to the national programs. ^4^ Percentage of people of the target age invited to participate in the screening. ^5^ Percentage of invited people that participated in the screening. ^6^ Regional or national differences.

## Data Availability

Data are contained within the article or supplementary material. The data presented in this study are available in Supplementary Tables S1–S6.

## Supplementary Materials

The following are available online at https://www.mdpi.com/2072-6694/13/5/1101/s1, Table S1: World and colorectal cancer in 2020. New cases, incidence and mortality rates estimation in continents. Obtained from Global Cancer Observatory (GBO) 2020, International Agency for Research on Cancer (http://gco.iarc.fr/ (accessed on 21 January 2021) [6]), World Health Organization, Table S2: World and colorectal cancer in 2020. New cases, incidence and mortality rates estimation in world areas. Obtained from Global Cancer Observatory (GBO) 2020, International Agency for Research on Cancer (http://gco.iarc.fr/ (accessed on 21 January 2021) [6]), World Health Organization, Table S3: Europe and colorectal cancer in 2020. New cases estimation, incidence and mortality rates in European countries for males and females. Obtained from Global Cancer Observatory (GBO) 2020, International Agency for Research on Cancer (http://gco.iarc.fr/ (accessed on 21 January 2021) [6]), World Health Organization, Table S4: Italy and colorectal cancer. New cases estimation, incidence and mortality for regions of Italy for males and females, Table S5: Colorectal cancer screening in Italy. Invitation rates of the target population and participation rates of the invited people in northern central and southern of Italy, Table S6: Colorectal cancer screening in Italy. Population, percentage of people older than 65 years and participation in FIT screening of the invited target population for each region of Italy.
