# Supplementary material for: The Roadmap of Colorectal Cancer Screening
Source: Cancers (Basel). 2021 Mar 4;13(5):1101. doi: 10.3390/cancers13051101 (PMC7961708; doi:10.3390/cancers13051101)
Supplement: Supplementary file 1 [file cancers-13-01101-s001.pdf]

*cancers*

*Supplementary Materials*

# The Roadmap of Colorectal Cancer Screening

**Enea Ferlizza *, Rossella Solmi, Michela Sgarzi, Luigi Ricciardiello and Mattia Lauriola**

## Supplementary

**Table S1.** World and colorectal cancer in 2020. New cases, incidence and mortality rates estimation in continents. Obtained from Global Cancer Observatory (GBO) 2020, International Agency for Research on Cancer (http://gco.iarc.fr/today), World Health Organization.

| Continents | New Cases | Incidence Crude Rate[1] | Incidence ASR[1] | Number of death | Mortality Crude Rate[1] | Incidence ASR[1] | M/I ratio[2] (%) |
|---|---|---|---|---|---|---|---|
| Africa | 66,198 | 4.9 | 8.4 | 42,875 | 3.2 | 5.6 | 64.8 |
| Asia | 1,009,400 | 21.8 | 17.6 | 506,449 | 10.9 | 8.6 | 50.2 |
| Europe | 519,820 | 69.4 | 30.4 | 244,824 | 32.7 | 12.3 | 47.1 |
| Latin America and the Caribbean | 134,943 | 20.6 | 16.6 | 69,435 | 10.6 | 8.2 | 51.5 |
| North America | 180,575 | 49 | 26.2 | 63,987 | 17.3 | 8.2 | 35.4 |
| Oceania | 20,654 | 48.4 | 29.8 | 7,603 | 17.8 | 9.3 | 36.8 |

[1] Crude and age standardized incidence and mortality rates (ASR) per 100,000 inhabitants. [2] M/I mortality to incidence ratio.

**Table S2.** World and colorectal cancer in 2020. New cases, incidence and mortality rates estimation in world areas. Obtained from Global Cancer Observatory (GBO) 2020, International Agency for Research on Cancer (http://gco.iarc.fr/today), World Health Organization.

| World Areas | Continents | New Cases | Incidence Crude Rate[1] | Incidence ASR[1] | Deaths | Morality Crude Rate[1] | Mortality ASR[1] | M/I ratio[2] (%) |
|---|---|---|---|---|---|---|---|---|
| Eastern Africa | Africa | 18,306 | 4.1 | 7.9 | 13,236 | 3 | 5.9 | 72.3 |
| Middle Africa | Africa | 5,767 | 3.2 | 6.8 | 4,228 | 2.4 | 5.2 | 73.3 |
| Northern Africa | Africa | 20,858 | 8.5 | 9.7 | 11,530 | 4.7 | 5.4 | 55.3 |
| Southern Africa | Africa | 7,684 | 11.4 | 13.7 | 3,943 | 5.8 | 7.2 | 51.3 |
| Western Africa | Africa | 13,583 | 3.4 | 6.7 | 9,938 | 2.5 | 5.1 | 73.2 |
| Caribbean | America | 11,454 | 26.3 | 18.2 | 6,983 | 16 | 10.4 | 61.0 |
| Central America | America | 19,535 | 10.9 | 10.4 | 10,439 | 5.8 | 5.5 | 53.4 |
| Northern America | America | 180,575 | 49 | 26.2 | 63,987 | 17.3 | 8.2 | 35.4 |
| South America | America | 103,954 | 24.1 | 18.5 | 52,013 | 12.1 | 8.9 | 50.0 |
| Eastern Asia | Asia | 757,849 | 45.2 | 25.9 | 368,072 | 21.9 | 11.8 | 48.6 |
| South-Central Asia | Asia | 102,987 | 5.1 | 5.5 | 59,206 | 2.9 | 3.2 | 57.5 |
| South-Eastern Asia | Asia | 106,995 | 16 | 14.8 | 57,064 | 8.5 | 7.9 | 53.3 |
| Western Asia | Asia | 41,569 | 14.9 | 16.8 | 22,107 | 7.9 | 8.9 | 53.2 |
| Central and Eastern Europe | Europe | 172,950 | 59 | 29.3 | 93,384 | 31.9 | 14.5 | 54.0 |
| Northern Europe | Europe | 81,638 | 76.8 | 33.6 | 33,768 | 31.8 | 11.4 | 41.4 |
| Southern Europe | Europe | 123,588 | 80.6 | 31.9 | 55,406 | 36.1 | 11.5 | 44.8 |
| Western Europe | Europe | 141,644 | 72.2 | 28.7 | 62,266 | 31.7 | 10.2 | 44.0 |
| Australia and New Zealand | Oceania | 19,644 | 64.8 | 33.2 | 7,038 | 23.2 | 9.5 | 35.8 |
| Melanesia | Oceania | 804 | 7.2 | 11.4 | 452 | 4.1 | 6.7 | 56.2 |
| Micronesia | Oceania | 93 | 16.9 | 16.6 | 53 | 9.7 | 9.5 | 57.0 |
| Polynesia | Oceania | 113 | 16.5 | 15.5 | 60 | 8.8 | 8.4 | 53.1 |

[1] Crude and age standardized incidence and mortality rates (ASR) per 100,000 inhabitants. [2] M/I mortality to incidence ratio.

**Table 3.** Europe and colorectal cancer in 2020. New cases estimation, incidence and mortality rates in European countries for males and females. Obtained from Global Cancer Observatory (GBO) 2020, International Agency for Research on Cancer (http://gco.iarc.fr/today), World Health Organization.

| | | **New cases[1]** | | | Incidence[2] | | | Mortality[2] | | |
|---|---|---|---|---|---|---|---|---|---|---|
| **ISO code** | **Country** | **Total** | **Males** | **Females** | Total | Males | Females | Total | Males | Females |
| ALB | Albania | 1858 | 922 | 936 | 7.7 | 8.8 | 6.8 | 3.8 | 4.4 | 3.4 |
| AUT | Austria | 12006 | 6088 | 5918 | 21 | 26.5 | 16.4 | 8.7 | 11.6 | 6.3 |
| BLR | Belarus | 8821 | 4076 | 4745 | 30.2 | 39.4 | 24.8 | 14.1 | 19.7 | 10.9 |
| BEL | Belgium | 19897 | 8163 | 11734 | 35.3 | 43.6 | 28 | 10 | 12.6 | 7.8 |
| BIH | Bosnia and Herzegovina | 3538 | 1984 | 1554 | 27 | 34.6 | 20.8 | 14.5 | 19.6 | 10.5 |
| BGR | Bulgaria | 9044 | 4983 | 4061 | 27.4 | 36.6 | 20.3 | 14.7 | 20.7 | 10.3 |
| HRV | Croatia | 5372 | 2478 | 2894 | 36.3 | 50.8 | 24.9 | 19.6 | 28.2 | 13.5 |
| CYP | Cyprus | 1582 | 821 | 761 | 24.3 | 35.6 | 14.3 | 10.7 | 14 | 7.9 |
| CZE | Czech Republic | 16840 | 9117 | 7723 | 33.7 | 44.4 | 25.2 | 12.3 | 17 | 8.6 |
| DNK | Denmark | 9843 | 4760 | 5083 | 40.9 | 47.1 | 35.6 | 11.8 | 13.7 | 10.2 |
| EST | Estonia | 2063 | 1228 | 835 | 28.3 | 35.6 | 23.9 | 13.8 | 18.6 | 11.1 |
| FIN | Finland | 10938 | 5710 | 5228 | 25.7 | 29.4 | 22.8 | 8.8 | 10.9 | 7.2 |
| FRA | France | 124153 | 66070 | 58083 | 30.1 | 36.3 | 24.9 | 10.4 | 13.3 | 8.1 |
| DEU | Germany | 137656 | 67959 | 69697 | 25.8 | 30.4 | 21.8 | 9.9 | 12.9 | 7.3 |
| GRC | Greece | 14558 | 6786 | 7772 | 26.9 | 34.4 | 20.5 | 10.7 | 14.1 | 7.8 |
| HUN | Hungary | 13799 | 6234 | 7565 | 45.3 | 62 | 33.1 | 20.2 | 29 | 14 |
| ISL | Iceland | 446 | 220 | 226 | 28.5 | 32.8 | 24.3 | 9.5 | 10.9 | 8.1 |
| IRL | Ireland | 7936 | 4503 | 3433 | 34.9 | 42.6 | 27.9 | 12.4 | 15.7 | 9.4 |
| ITA | Italy | 94450 | 39317 | 55133 | 29.3 | 34.2 | 25.2 | 10.1 | 12.7 | 8.1 |
| LVA | Latvia | 2794 | 1531 | 1263 | 36.8 | 48.8 | 30.1 | 12.3 | 15.9 | 10.4 |
| LTU | Lithuania | 4006 | 2237 | 1769 | 27.6 | 36.4 | 22.3 | 11.7 | 16.1 | 9.4 |
| LUX | Luxembourg | 889 | 392 | 497 | 26.3 | 29.7 | 23.7 | 8.7 | 11.2 | 6.4 |
| MKD | Macedonia | 1880 | 892 | 988 | 26.1 | 26.6 | 26.1 | 13 | 13.9 | 12.3 |
| MLT | Malta | 739 | 336 | 403 | 25.7 | 31.1 | 21.2 | 10.1 | 11.9 | 8.6 |
| MDA | Moldova Republic | 2770 | 1278 | 1492 | 30 | 44.3 | 19.7 | 17.6 | 26.7 | 11.3 |
| MNE | Montenegro | 696 | 318 | 378 | 27.4 | 35.2 | 21.1 | 13.7 | 21.5 | 7.8 |
| NLD | The Netherlands | 30305 | 14580 | 15725 | 41 | 48.4 | 34.3 | 13.5 | 16.2 | 11.1 |
| NOR | Norway | 8899 | 5229 | 3670 | 41.9 | 45.4 | 38.7 | 13.5 | 15.1 | 12.1 |
| POL | Poland | 42921 | 18277 | 24644 | 30.5 | 41.7 | 21.9 | 16.1 | 22.8 | 11.3 |
| PRT | Portugal | 13800 | 6759 | 7041 | 39.4 | 55.2 | 26.6 | 13 | 18.6 | 8.8 |
| ROU | Romania | 21115 | 9030 | 12085 | 26.7 | 36.3 | 19.3 | 14.8 | 21.1 | 10.2 |
| RUS | Russian Federation | 126046 | 50994 | 75052 | 27.8 | 34.4 | 23.9 | 13.9 | 18.6 | 11.3 |
| SRB | Serbia | 12133 | 5409 | 6724 | 33.6 | 46.4 | 22.8 | 16.7 | 23.7 | 11.1 |
| SVK | Slovakia | 5931 | 2853 | 3078 | 43.9 | 60.7 | 31.1 | 21 | 29.6 | 14.8 |
| SVN | Slovenia | 3244 | 1834 | 1410 | 39.6 | 55.8 | 25.4 | 11.7 | 16.1 | 8.4 |
| ESP | Spain | 68701 | 34613 | 34088 | 35.8 | 47.7 | 25.4 | 11.5 | 15.5 | 8.2 |
| SWE | Sweden | 18483 | 10949 | 7534 | 27.8 | 30.5 | 25.2 | 10.8 | 12.1 | 9.7 |
| CHE | Switzerland | 14240 | 6948 | 7292 | 22.3 | 25.7 | 19.4 | 7.5 | 9.1 | 6.2 |
| UKR | Ukraine | 31180 | 12917 | 18263 | 25.5 | 33.6 | 20.5 | 12.9 | 18.1 | 9.9 |
| GBR | United Kingdom | 110669 | 56780 | 53889 | 34.1 | 40 | 29 | 11.4 | 13.5 | 9.6 |

[1] Estimated number of new cases in 2020. [2] Age standardized incidence and mortality rates for 100,000 inhabitants (world).

**Table 4.** Italy and colorectal cancer. New cases estimation, incidence and mortality for regions of Italy from males and females.

| | | New cases [1] | | | Incidence [2] | | Mortality [3] | | |
|---|---|---|---|---|---|---|---|---|---|
| | **Regions** | **Total** | **M** | **F** | **M** | **F** | **Total** | **M** | **F** |
| NORTH | Emilia Romagna | 3600 | 2000 | 1600 | 73.9 | 49.8 | 32.2 | 33.9 | 30.6 |
| | Friuli Venezia Giulia | 1500 | 900 | 600 | 126.6 | 70.9 | 38.2 | 42.4 | 34.2 |
| | Liguria | 1750 | 950 | 800 | 94.6 | 61.5 | 44.7 | 52.4 | 37.6 |
| | Lombardia | 7100 | 4100 | 3000 | 67 | 43.3 | 30.0 | 33.3 | 26.9 |
| | Piemonte | 4000 | 2200 | 1800 | 82.9 | 61.5 | 38.0 | 41.8 | 34.4 |
| | Trentino Alto Adige | 900 | 500 | 400 | 82.8 | 55.2 | 24.7 | 27.0 | 22.5 |
| | Valle d'Aosta | <100 | < 50 | < 50 | 64.5 | 46.7 | 33.4 | 26.0 | 40.4 |
| | Veneto | 3900 | 2100 | 1800 | 78.1 | 53.9 | 31.5 | 34.1 | 29.1 |
| CENTER | Lazio | 5200 | 2700 | 2500 | 88.5 | 65.8 | 32.3 | 36.2 | 28.7 |
| | Marche | 1150 | 650 | 500 | 87.2 | 63.5 | 33.8 | 39.1 | 28.7 |
| | Toscana | 3600 | 2000 | 1600 | 86.7 | 61.4 | 33.2 | 36.5 | 30.2 |
| | Umbria | 850 | 450 | 400 | 82.5 | 57.1 | 34.4 | 41.4 | 27.9 |
| SOUTH | Abruzzo | 1100 | 600 | 500 | 83.9 | 55.9 | 33.3 | 40.9 | 26.0 |
| | Basilicata | 450 | 250 | 200 | 80 | 47.7 | 30.3 | 33.5 | 27.2 |
| | Calabria | 1400 | 800 | 600 | 79.2 | 48.5 | 31.4 | 37.6 | 25.4 |
| | Campania | 3800 | 2200 | 1600 | 82.8 | 50.9 | 26.8 | 30.9 | 22.9 |
| | Molise | 250 | 150 | 100 | 83.9 | 55.9 | 34.2 | 41.0 | 27.6 |
| | Puglia | 2900 | 1800 | 1100 | 81 | 45.8 | 28.4 | 32.6 | 24.4 |
| | Sardegna | 1500 | 1000 | 500 | 113 | 36.6 | 36.0 | 42.6 | 29.5 |
| | Sicilia | 3950 | 2100 | 1850 | 84.6 | 59.5 | 32.7 | 36.4 | 29.1 |
| | Italy | 48900 | 27450 | 21450 | 85.2 | 54.6 | 32.0 | 35.9 | 28.4 |

[1] Estimated number of new cases for the year 2019; [2] Crude incidence rates estimation for 100,000 inhabitants for the year 2019; [3] Crude mortality rates for 100,000 inhabitants for the year 2018 (Italian National Institute of Statistics, ISTAT).

**Table 5.** CRC screening in Italy. Invitation rates of the target population and participation rates of the invited people in north center and south of Italy.

| | Invitation Rate (%) | Participation Rate (%) |
|---|---|---|
| North | 90 | 60 |
| Centre | 96 | 45 |
| South-Isles | 44 | 23 |
| Italy | 75 | 42 |

**Table 6.** CRC screening in Italy. Population, percentage of people older than 65 years and participation to FIT screening of the invited target population for each region of Italy.

| | Regions | Inhabitants | > 65 years (%) | Participation (%) |
|---|---|---|---|---|
| NORTH | Piemonte | 4,375,865 | 25.3 | nd |
| | Val D'Aosta | 126,202 | 23.5 | 63.6 |
| | Liguria | 1,067,648 | 28.4 | 40.2 |
| | Lombardia | 10,036,258 | 22.4 | 66.7 |
| | Trentino Alto Adige | 1,215,538 | 20.6 | 57.5 |
| | Veneto | 4,905,037 | 22.6 | 65.9 |
| | Friuli Venezia Giulia | 1,556,981 | 25.9 | 65.2 |
| | Emilia Romagna | 4,452,629 | 23.8 | 64.0 |
| CENTER | Toscana | 3,736,968 | 25.2 | 58.9 |
| | Umbria | 884,640 | 25.2 | 53.0 |
| | Marche | 1,531,753 | 24.5 | 47.0 |
| | Lazio | 5,896,693 | 21.4 | 21.6 |
| SOUTH | Abruzzo | 1,315,196 | 23.6 | 20.3 |
| | Molise | 308,493 | 24.2 | 43.3 |
| | Campania | 5,826,860 | 18.5 | 13.3 |
| | Puglia | 4,048,242 | 21.7 | 4.6 |
| | Basilicata | 567,118 | 22.6 | 35.5 |
| | Calabria | 1,956,687 | 21.2 | 5.6 |
| | Sicilia | 5,026,989 | 20.8 | 22.9 |
| | Sardegna | 1,648,176 | 23.2 | 34.6 |
| ITALY | Total | 60,483,973 | 23.2 | 42.2 |
